# An Efficient Method for the Genetic Transformation of *Acmella oleracea* L. (*Spilanthes acmella* Linn.) with *Agrobacterium tumefaciens*

**DOI:** 10.3390/plants10020198

**Published:** 2021-01-21

**Authors:** Valentina Maggini, Priscilla Bettini, Fabio Firenzuoli, Patrizia Bogani

**Affiliations:** 1 Research and Innovation Center in Phytotherapy and Integrated Medicine (CERFIT), Careggi University Hospital, Via delle Oblate 4, 50141 Florence, Italy; fabio.firenzuoli@unifi.it; 2 Department of Biology, University of Florence, Via Madonna del Piano 6, Sesto Fiorentino, 50019 Florence, Italy; priscilla.bettini@unifi.it (P.B.); patrizia.bogani@unifi.it (P.B.)

**Keywords:** *Acmella oleracea*, *Agrobacterium*, genetic transformation, stem node regeneration, kanamycin resistance, GUS detection

## Abstract

*Acmella oleracea* L. is an important medicinal plant, commonly known as the toothache plant. It is a rich source of secondary metabolites used for the treatment of different human disorders. The demand for *Acmella oleracea* L. has increased due to its putative health benefits (in terms of both biomass quantity and bioactive compound purification). In vitro plant cultures have allowed the rapid increase of raw material availability through the use of suitable regeneration and multiplication systems. On the other hand, there is a general lack of methods for *Acmella* genetic transformation as a promising new technological approach for the improvement of secondary metabolites. In this work, an efficient transformation protocol has been established using the *Agrobacterium tumefaciens* LBA4404 strain bearing the binary vector pBI121 containing the *NPTII* gene for the resistance to kanamycin. Plant genetic transformation has been verified by direct polymerase chain reaction and GUS assay on regenerants. Transformation efficiency has been affected by the high level of the selection agent kanamycin. To our knowledge, this is the first report on the genetic transformation of *A. oleracea*, paving the way to further studies to improve in vitro plant growth and secondary metabolite production.

## 1. Introduction

*Acmella oleracea* (L.) R.K. Jansen [1], also known as *Spilanthes acmella* Murr. [2,3,4] or *Spilanthes oleracea* L. [5], is part of the Asteraceae family. It was first discovered in Peru and is now commonly found in tropical and subtropical regions across the world, especially in the north of Brazil, where it is known as jambu [6,7,8,9]. Generally, this plant is an annual or perennial herb of short duration and reaches a height ranging from 20 to 90 cm. The leaves are of simple origin, oval, with irregular edges. The stem is cylindrical and moderately plastic. The typical flower color is generally yellow, with a red tip, and dome-shaped, with well-arranged sepals surrounded by small petals [10]. It is an important medicinal plant, traditionally used for its analgesic and anti-inflammatory properties (mainly for toothache and oral cavity inflammation treatments), but also for its recognized antipyretic, anticonvulsant, antidiarrheal, antidiuretic, antiseptic, anti-fungal, anti-protozoal, and insecticidal properties. Moreover, it is used for culinary purposes [7,8,10,11]. These properties are a result of its endogenous content, of bioactive compounds, such as sterols, coumarins, flavonoids, saponins, terpenoids, polysaccharides, and, in particular, alkylamides [7,8,12]. Among the alkylamides, spilanthol ((E, E, Z)-2,6,8-decatrienoic acid N-isobutylamide) is considered to be the most potent bioactive compound found in *A. oleracea*. First identified by Gerber in 1903 as identical to affinin 1, spilanthol has been mostly found in *Acmella* flowers, leaves, and stems [4,7,8,12,13,14,15,16], but also in roots [17,18], and its accumulation in in vitro cell cultures has been documented [2,3]. Due to its pharmacological importance, based on a series of effects typical of alkylamides, such as analgesic, neuroprotective, antioxidant, antimutagenic, anti-cancer, anti-inflammatory, antimicrobial, anti-larvicidal, and insecticidal activities [19], many protocols for spilanthol production and extraction have been developed (see [19] for a review; [20]). Besides recent in vivo treatments with biostimulants [21], a good approach to obtain a great number of plants, in a short period of time under selected standardized conditions of cultivation, is the use of in vitro plant cultures [2,3,8,22,23,24,25,26,27]. These strategies have allowed rapid improvement of raw material and metabolite production. On the other hand, although their importance in many medicinal species is well known, methods for *Acmella* genetic transformation are lacking. Hairy roots, for example, have proven to be a promising tool for the increased production of secondary metabolites in hundreds of traditional herbs [28,29,30]. Moreover, recent developments in metabolic engineering techniques have made possible the introduction of novel biosynthetic pathways in both medicinal and commercial crops to enhance their market and nutritional value [31,32]. *Agrobacterium tumefaciens* is so far the most used and powerful tool for the introduction of a series of candidate genes and transcriptional regulators in medicinal plants [33,34]. Therefore, since, to our knowledge, there is no data in the literature on the genetic transformation of *Acmella* spp., in this study, we report the development of the first successful *Agrobacterium*-mediated transformation protocol for the integration and expression of *NPTII* and *GUS* genes.

## 2. Results and Discussion

### 2.1. Explant Effect on the Regeneration by Organogenesis in A. oleracea

**Figure 1:**
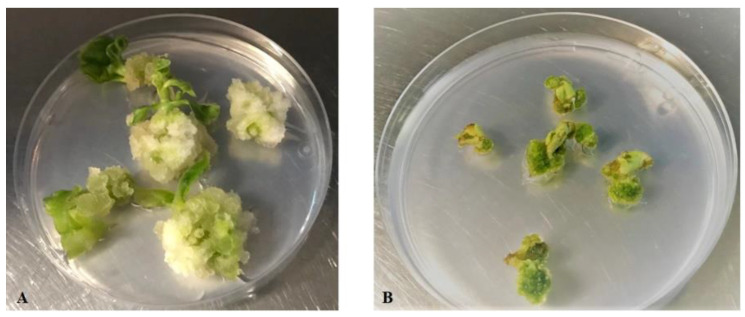
Morphogenetic behavior of *A. oleracea* nodal (**A**) and leaf (**B**) explants after 30 days of culture on LS medium supplemented with 1 mg/L 6-Benzylaminopurine (BAP) and 0.1 mg/L 1-Naphthaleneacetic acid (NAA) (LS1 regeneration medium).

Due to increasing market demand, efficient regeneration systems have been reported in *A. oleracea*. Different established protocols showed that the best hormone combination to induce shoots formation on in vitro and in vivo explants was based on the ratio between 6-Benzylaminopurine (BAP) and 1-Naphthaleneacetic acid (NAA) at different concentrations. Moreover, leaf explants regenerated multiple shoots from in vitro seedlings, and shoot differentiation was better from in vivo nodes than other tissue explants [2,3,23,35,36,37,38]. In our work, we used Linsmaier & Skoog (LS) medium supplemented with 1 mg/L BAP and 0.1 mg/L NAA (LS1) to induce shoot proliferation on leaf and nodal explants derived from in vitro 2-month-old *Acmella* axenic plants. Callus and shoot differentiation was observed on 100% of nodal stem explants (*n* = 60), starting from the fourth day of culture on LS1 regeneration medium (Figure 1A). After 30 days of cultivation, the average number of 0.5 cm high shoots per explant was equal to three. Leaf explants (and vice versa) were capable of only proliferating callus. All of the leaf explants showed callus dedifferentiation after 30 days of culture (Figure 1B), and they never produced any seedlings, even after two further transfers on fresh LS1 medium at intervals of 30 days each.

Thus, nodal stems were chosen as sources of explants for transformation of *A. oleracea* with the *A. tumefaciens* pBI121 vector, containing the neomycin phosphotransferase *NPTII* gene for resistance to kanamycin, the selection agent more frequently used in *Agrobacterium*-mediated plant transformation experiments.

### 2.2. Agrobacterium-Mediated Transformation

#### 2.2.1. Determination of a Kanamycin Dose–Response Curve for the Selection of Acmella pBI121 Transformants

**Figure 2:**
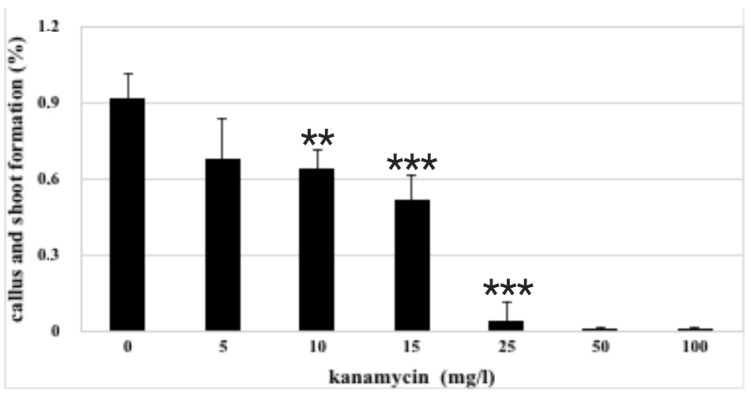
Inhibitory effect of kanamycin on callus and shoot formation from *A. oleracea* nodal explants after 30 days of culture on LS1 regeneration medium. *n* = 5; error bars represent standard deviations referred to five replicates; ** *p* ≥ 0.01; *** *p* ≥ 0.0001.

In order to evaluate the effect of kanamycin on shoot differentiation, a dose–response curve was planned. Results of the experiments (Figure 2) showed a dose-dependent toxic effect of kanamycin on callus and shoot formation, significantly inhibited from 10 to 25 mg/L (extremely toxic dose), and completely suppressed at 50 mg/L. An antibiotic concentration of 10 mg/L was therefore chosen for *A. oleracea* transformation.

#### 2.2.2. Nodal Explant Infection

**Figure 3:**
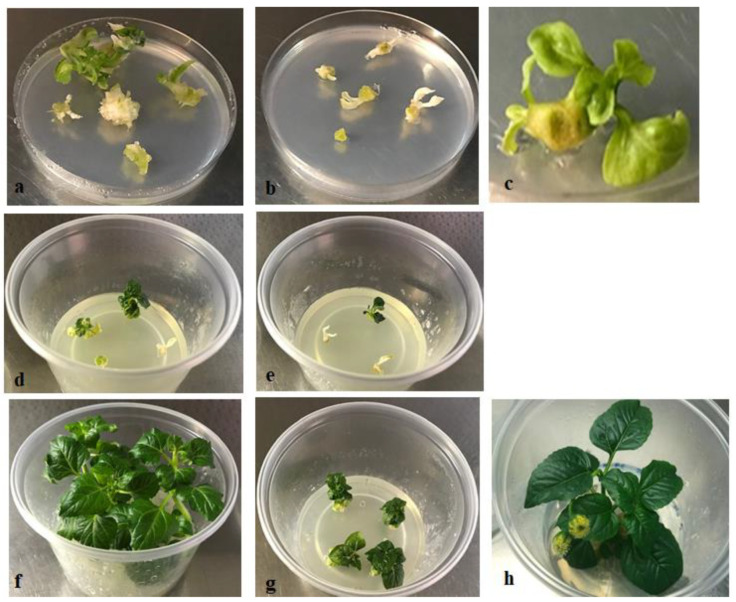
Plant transformation in *Acmella oleracea*. (**a,b**) Nodal explants infected with *Agrobacterium* LBA4404::pBI121 showing regenerated shoots after 30 days of culture on LS1 medium with the addition of 10 mg/L kanamycin and 500 mg/L cefotaxime. (**c**) Control (not infected) nodal explants on the same selection medium 30 days after co-cultivation with *Agrobacterium*. (**d**,**e**) Isolated putative transgenic shoots 40 days after co-cultivation with *Agrobacterium* on LS1 selection medium. (**f**) *Acmella* plantlets isolated from control nodal explants after two months of growth on LS medium devoid of hormones; *Acmella* transgenic shoots after two (**g**) and four months (**h**) of growth on LS selection medium containing 10 mg/L kanamycin.

Thirty nodal stem explants were infected with *A. tumefaciens* LBA4404 strain harboring the pBI121 vector and 30 were used as not infected controls. After 96 h of co-cultivation, all of the infected explants and half of the non-infected ones were transferred to LS1 selection medium with kanamycin (10 mg/L, initial selection dose) and cefotaxime (500 mg/L). Fifteen control explants were placed on plates containing LS1 medium without antibiotics. After 30 days, 92% of the infected explants, and 25% of controls, showed callus and shoot regeneration (Figure 3a–c). The presence of shoots on control explants indicated the occurrence of escape from selection at the chosen kanamycin concentration. However, under further selection, the same control regenerated shoots appeared bleached, less vigorous, and stopped their growth after only one transfer on medium with the selection agent. After 40 days from co-cultivation, 23 independent putative transgenics shoots, about 0.5 cm in length, were isolated and transferred to Wavin containers (Lab Associates B.V., The Netherlands) with half strength LS selection medium containing 2% sucrose and devoid of hormones, to allow root formation and shoot elongation (Figure 3d,e). Only 7 out of 23 shoots survived the selection, and after two months from their isolation showed a reduced growth (Figure 3g) compared to the controls (Figure 3f).

### 2.3. Molecular Analysis of Putative Transformants

#### 2.3.1. Analysis of the Presence of Transgenes by Direct PCR

Before the molecular analysis of each putative transformant, a sterility test was performed to exclude the presence of *Agrobacterium* inside the leaf plant tissues used as templates in experiments of direct PCR. Results, reported in Figure S1, clearly showed the absence of *Agrobacterium* growth in yeast extract-peptone (YEP) medium (supplemented with rifampicin and kanamycin, antibiotics specific for *Agrobacterium* and its vector, respectively) containing leaf macerates from both putative transformed and untransformed control shoots, as evidenced by the lack of turbidity of the solutions. This method has proven very useful at checking the persistence of Agrobacterium in tobacco transgenic plants [39]. However, it is well-established in literature that cefotaxime concentration of 500 mg/L (used in our work) completely eliminates the *Agrobacterium* presence after two weeks from the infection of explants (see for instance [40,41,42]). Thus, we performed the test on eleven-week-old plants after the isolation from the infected explants, grown on selection medium containing kanamycin and cefotaxime. An alternative time-saving potentiality to test the absence of *Agrobacterium* could be the PCR amplification with specific primers for marker genes present on Ti helper plasmid (for instance, *vir* genes) or on *Agrobacterium* chromosome (e.g., *chv* genes).

**Figure 4:**
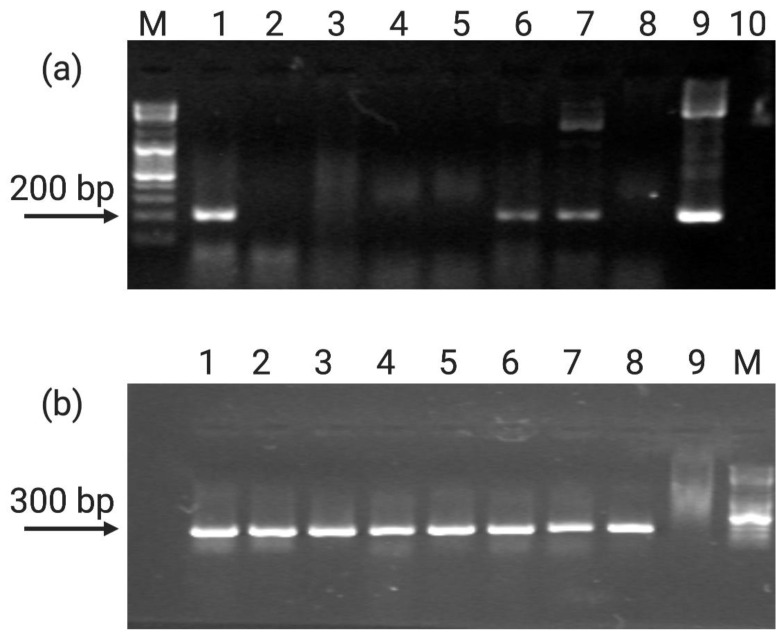
Electrophoretic profile showing the presence of *GUS* amplified fragment (215 bp) (**a**) and the housekeeping chloroplastic gene (**b**) in transformed *A. oleracea* shoots. (**a**)M: Gene Ruler DNA ladder mix (Thermo Scientific); 1–7: *A. oleracea* transformants; 8: *A. oleracea* wild type control; 9: plasmid DNA as positive control; 10: PCR negative control. (**b**) 1–7: *A. oleracea* transformants; 8: *A. oleracea* wild type control; 9: PCR negative control; M: Gene Ruler DNA ladder mix.

Subsequently, to demonstrate the presence of transgenes in putative *A. oleracea* transformants, a direct PCR was performed by using a pair of primers amplifying a region of the *GUS* gene (Table S1). Moreover, a pair of universal primers specific of a chloroplastic region 297 bp long was used as internal control of the direct PCR reaction. Both the reactions were performed at 62 °C, the recommended annealing temperature. Results shown in Figure 4 evidenced the presence of the *GUS* gene only in three regenerated shoots and in the plasmid DNA used as positive control (Figure 4a), while the chloroplastic band was present in all of the samples, suggesting optimal PCR conditions (Figure 4b).

After four months of culture under selection pressure, three *GUS* positive genotypes (pBI121 1, 6, and 7 transgenic lines) showed an in vitro growth as the wild type ones. Interestingly, in vitro flowering occurred in one of these genotypes (Figure 3h). They were then successfully grown in presence of increasing concentrations of kanamycin, namely the inhibitory doses 25, 50, and 100 mg/L, and further analyzed for *GUS* expression. Figure S2 shows individual plants from each transgenic line subcultured on half strength LS medium supplemented with 100 mg/L kanamycin.

As evidenced from the dose–response curve (Section 2.2.1), the use of 25 mg/L kanamycin resulted extremely toxic during shoot differentiation. Remarkably, after genetic transformation, the putative transgenic regenerants were also able to grow on higher levels of kanamycin up to 100 mg/L, confirming the efficacy of the reported method.

#### 2.3.2. *GUS* Gene Expression Analysis

**Figure 5:**
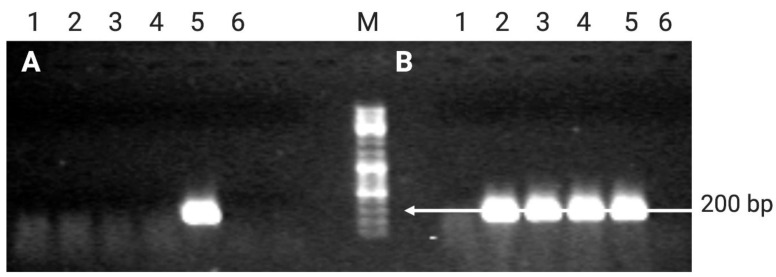
RT-PCR amplification on total RNA of (**A**) *CaMV* promoter and (**B**) *GUS* gene sequence from transformed and untransformed *A. oleracea* regenerated plants. 1: *A. oleracea* untransformed control; 2: *A. oleracea* pBI121_1 transgenic line; 3: *A. oleracea* pBI121_6 transgenic line; 4: *A. oleracea* pBI121_7 transgenic line; 5: positive control (pBI121 plasmid DNAl); 6: negative control (RT-PCR reaction without RNA); M: Gene Ruler DNA ladder mix (Thermo Scientific).

*GUS* transcription was demonstrated in the 4-month-old selected transgenic plants by RT-PCR. The presence of genomic DNA contamination in the same RNA samples had been ruled out by amplification of the *CaMV* promoter region. No amplification was detected in the untransformed control, transgenic samples, and negative control (RT-PCR reaction lacking RNA). Amplification only resulted for pBI121 plasmid DNA used as positive control (Figure 5A). The amplification with GusFw and GusRev primers produced the expected 215 bp long fragment (Figure 5B).

**Figure 6:**
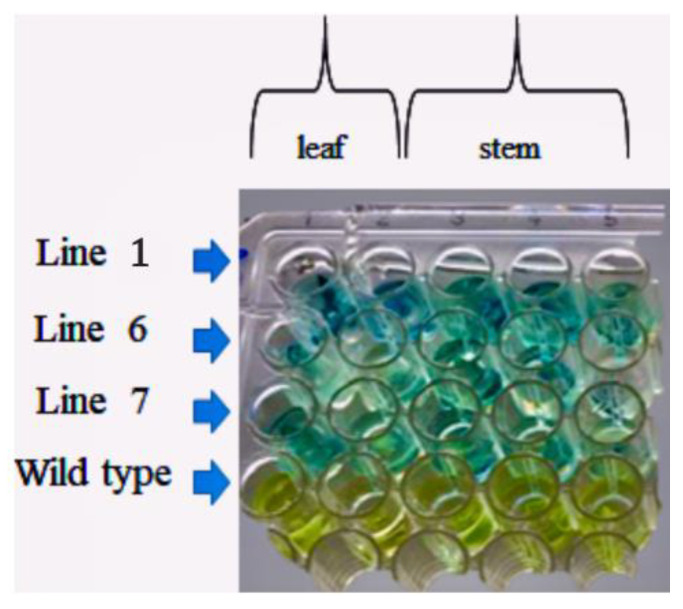
In vivo *GUS* assay on leaf and stem explants of *A. oleracea* transgenic and wild type regenerated plants.

RNA quality was checked by the RT-PCR amplification of the chloroplastic *PSBA* housekeeping gene (Figure S3). The amplification was carried out with the primers pair psbAFW and psbAREV (Table S1) designed on the sequence of the *Nicotiana tabacum* gene [43] and produced the expected 942 bp fragment in all samples. Coherently, in vivo GUS assay showed the characteristic blue color of the X-Gluc reaction in all transgenic lines tissues, indicating high GUS activity (Figure 6).

## 3. Materials and Methods

### 3.1. Plant Material and In Vitro Culture Establishment

In vitro seedlings of *A. oleracea* were obtained from seeds (Saflax–Frank Laue, Münster, Germany); surface was sterilized using 5% (*v/v*) hypochlorite containing a drop of Tween 20 and washed three times for 10 min in sterile distilled water. Surface-sterile seeds were then germinated on LS medium [44] containing 30 g/L of sucrose (Lab Associates B.V., Oudenbosch, The Netherlands) and 0.7% of Phyto agar (Duchefa, Amsterdam, The Netherlands), and further grown in a growth chamber at 24 ± 1 °C under a 16 h light/8 h dark lighting condition. *Acmella* shoots were maintained in vitro by sub-culturing the upper part of the shoots on fresh LS medium at 20 days-intervals.

### 3.2. Regeneration by Organogenesis of A. oleracea

For shoot regeneration, axenic leaf and stem nodal explants 0.7–1.5 cm in size were cultivated on LS medium supplemented with 1 mg/L 6-Benzylaminopurine (BAP, Sigma-Aldrich, Dordrecht, The Netherlands) and 0.1 mg/L 1-Naphthaleneacetic acid (NAA, Sigma-Aldrich, Dordrecht, The Netherlands) (LS1 medium) at 24 ± 1 °C under a 16 h light/8 h dark lighting condition until shoot formation. For shoot elongation and root induction, each shoot 0.5 cm high was transferred on half strength LS medium without hormones and containing 2% sucrose. Each regeneration experiment consisted of 20 explants and was repeated three times.

### 3.3. Agrobacterium-Mediated Transformation of A. oleracea

#### 3.3.1. Determination of Kanamycin Concentration for the Selection of Transformants

To identify the optimal dose of kanamycin for the selection of putative transformants, the toxic effect of the antibiotic was verified at increasing concentrations from 5 to 10, 15, 25, 50, and 100 mg/L. To this aim, five leaf and five nodal explants per dose were prepared and sown on LS1 medium with kanamycin as described. The experiment was repeated five times, for a total of 25 explants for each dose. For the preparation of leaf explants, a sterile 7 mm diameter cork borer was used; 1.5 cm nodal explants were obtained by cutting the stems with a sterile scalpel. After 4 weeks of culture in a growth chamber at 24 ± 1 °C, the explant differentiation capability was recorded.

#### 3.3.2. Leaf Disc Transformation and Molecular Analysis of Transformants

*Agrobacterium tumefaciens* strain LBA4404 [45] containing the binary vector pBI121 [46] was used to transform the nodal explants of *A. oleracea* L. with the leaf disc infection technique [47]. A single colony of the *Agrobacterium* strain was inoculated in 3 mL of liquid YEB medium supplemented with 50 mg/L kanamycin (selecting for the pBI121 vector) and 20 mg/L rifampicin (selecting for LBA4404 *Agrobacterium* strain), and grown in agitation at 28 °C, overnight. One ml of the overnight suspension culture was then inoculated in 100 mL of fresh YEB medium, in presence of antibiotics and 100 μM acetosyringone (Sigma-Aldrich, Dordrecht, The Netherlands), and kept at 28 °C until OD_600_ = 1. Bacterial cells were then centrifuged at 10,000 rpm for 1 min and re-suspended in the same volume of LS basal medium. A 1:10 (*v*/*v*) dilution of the bacterial suspension in LS medium was then used for co-cultivation. Nodal explants were dipped into the co-cultivation medium for 40 min, blotted on sterile filter paper and then placed on Petri dishes (ten per dish) containing LS1 medium with 10 mg/L kanamycin as selection dose. Plates were maintained in the dark at 24 ± 1 °C for 96 h for co-cultivation. For transgenic shoot regeneration, the explants were transferred on LS1 containing 10 mg/L kanamycin and 500 mg/L cefotaxime under a 16-h light photoperiod. Primary transformants were isolated after five weeks of culture and placed on a hormone-free selection medium containing 10 mg/L kanamycin and decreasing concentrations of cefotaxime (from 500 to 300 mg/L) until the complete *Agrobacterium* elimination. Two independent experiments were carried out leading to the transformation of 30 nodal explants.

Before carrying out the molecular analysis by direct PCR, a sterility test was made to exclude the possibility that *Agrobacterium* was still present inside the intercellular spaces of putative transformed shoots. The sterility test is based on the absence of *Agrobacterium* growth in 3 mL of YEP medium supplemented with 20 mg/L rifampicin, 50 mg/L kanamycin, and 100 mL of leaf macerate obtained from each regenerated plant. To obtain the leaf macerate, an apical and a basal leaf from each individual shoot were crushed in 500 μL of 0.9% NaCl saline solution with a sterile pestle inside a sterile Eppendorf tube. The obtained macerates were then briefly centrifuged at 3000 rpm to remove the leaf debris. One hundred μ.L of supernatant were then inoculated in YEP medium containing the antibiotics. As positive control, 100 μL of a 0.1 OD_600_ *Agrobacterium* suspension culture were inoculated in the same culture medium. The solutions were incubated at 28 °C in agitation and in the dark for 72–96 h and observed for the eventual bacterial growth.

Transformation was confirmed using the Phire Plant Direct PCR Kit (Thermo Fisher Scientific™, Waltham, MA, USA) in combination with a pair of primers (Table S1) able to amplify a region of the *GUS* gene present on the pBI121 T-DNA (GenBank accession number AF485783.1). Moreover, the universal primer mix provided by the kit was used to amplify a chloroplastic region 298 bp long as positive control. The calculated annealing temperature for all primer pairs was 62 °C, according to the manufacturer’s instructions.

#### 3.3.3. *GUS* Assay with X-Gluc and *GUS* Expression Analysis with RT-PCR

For *GUS* assay, stem and leaf samples from each isolated putative transgenic shoot were collected and dipped for fifteen min in 500 μL of 100 mM phosphate buffer pH 7.2, containing 1 mM X-Gluc (5-bromo-4-chloro-3-indolyl-β-d-glucuronic acid) in N-N-dimethylformamide. Then, buffered X-Gluc was changed once and the samples were kept in an incubator at 37 °C until color development. Samples were washed twice with 95% ethanol to bleach and better observe the color development.

To assess the expression of the *GUS* gene in *Acmella GUS* (+) transgenic lines, total RNA of three selected genotypes and wild type not transformed plantlets was extracted from the leaves following the instructions of the GeneJet Plant RNA purification mini kit (Life Technologies, Monza, Italy). DNA contamination was removed with the Thermo Scientific RapidOut DNA Removal kit (Life Technologies, Monza, Italy). RNA concentration was estimated by fluorometry using the Qubit quantitation platform by Invitrogen. RT-PCR for the analysis of transgene expression was performed with the OneStep RT-PCR Kit (Qiagen, Venlo, The Netherlands) following the manufacturer’s guidelines. RT-PCR amplification of the *GUS* transgene was performed on total RNA using GusFw and GusRev primers (Table S1).

### 3.4. Statistical Data Analysis

The analysis of variance between kanamycin treatments was performed using One-way ANOVA (*p* < 0.05). For each experiment, 25 explants were sown (five explants per treatment, five replicates per treatment), and the experiment was repeated three times. Mean separations were performed using the method of Tukey. For the analysis, the PAST program version 1.89 was used [48].

## 4. Conclusions

In this work, we established a fast and efficient protocol for the genetic transformation of the emergent medicinal plant *Acmella oleracea*. The choice of nodal explants as source for the infection with *Agrobacterium* LBA4404::pBI121 and the use of 10 mg/L of kanamycin as selection agent were proven effective. Despite the presence of escape, the initially low antibiotic dose has successfully permitted both the selection and the regeneration of transformants, as confirmed by molecular techniques: real genetically transformed plantlets have then been stabilized under high selection pressure conditions. Moreover, the efficiency of transformation could be reduced by the presence of a mutation in the NPTII gene of pBI121 binary vector [46]; thus, other vectors with no mutations can be tested in order to increase the transformation efficiency in *A. oleracea*. Finally, a large number of nodal explants will allow obtaining a high number of recombinant shoots in only three months.

To our knowledge, this is the first report on the genetic transformation of *A. oleracea*. It could be performed with specific candidate genes for the production or the identification of novel bioactive compounds [31,32].

## Data Availability

All data reported here is available from the authors upon request.

## Supplementary Materials

The following are available online at https://www.mdpi.com/2223-7747/10/2/198/s1, Figure S1: Sterility test on *Acmella* transgenic shoots after 40 days from isolation and cultivation on LS selection medium with 10 mg/L kanamycin. 1–2: YEP medium with 100 mL of leaf macerates from two transgenic shoots, as an example; 3: YEP medium with 100 mL of leaf macerates from untransformed shoots; 4: YEP medium with 100 mL of saline solution (negative control); 5: YEP medium inoculated with LBA4404::pBI121 vector (positive control); Figure S2: Individual transgenic lines grown on half strength LS medium supplemented with 100 mg/L kanamycin; Figure S3: RT-PCR amplification of the *PSBA* chloroplastic gene on total RNA from wild type (untransformed) and transformed with pBI121 binary vector *A. oleracea* plants; Table S1: List of primer pairs used for Phire PCR and RT-PCR experiments.
