# Supplementary material for: An Efficient Method for the Genetic Transformation of Acmella oleracea L. (Spilanthes acmella Linn.) with Agrobacterium tumefaciens
Source: Plants (Basel). 2021 Jan 21;10(2):198. doi: 10.3390/plants10020198 (PMC7911432; doi:10.3390/plants10020198)
Supplement: Supplementary file 1 [file plants-10-00198-s001.pdf]

Figure S1: Sterility test on *Acmella* transgenic shoots after 40 days from isolation and cultivation on LS selection medium with 10 mg/l kanamycin. 1-2: YEP medium with 100 µl of leaf macerates from two transgenic shoots, as an example; 3: YEP medium with 100 µl of leaf macerates from untrasformed shoots; 4: YEP medium with 100 µl of saline solution (negative control); 5: YEP medium inoculated with LBA4404::pBI121 vector (positive control).

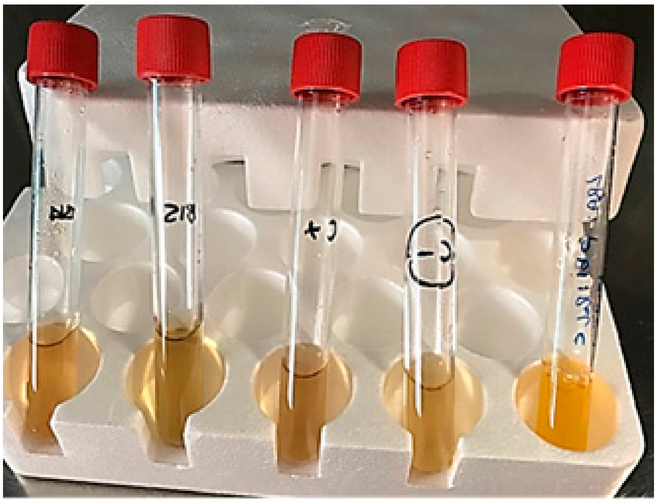

1 2 3 4 5

Figure S2. Individual transgenic lines grown on half strength LS medium supplemented with 100 mg/l kanamycin. A: line 1; B: line 6; C: line 7.

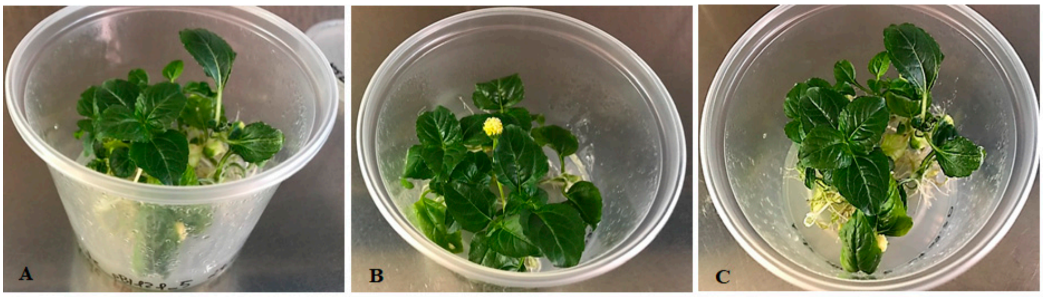

Figure S3. RT-PCR amplification of the *PSB*A chloroplastic gene on total RNA from wild type (untransformed) and transformed with pBI121 binary vector *A. oleracea* plants. 1: wild type untransformed control; 2-4: pBI121 transgenic *A. oleracea* plants; C-: PCR negative control; M: Gene Ruler ladder mix (Thermo Scientific).

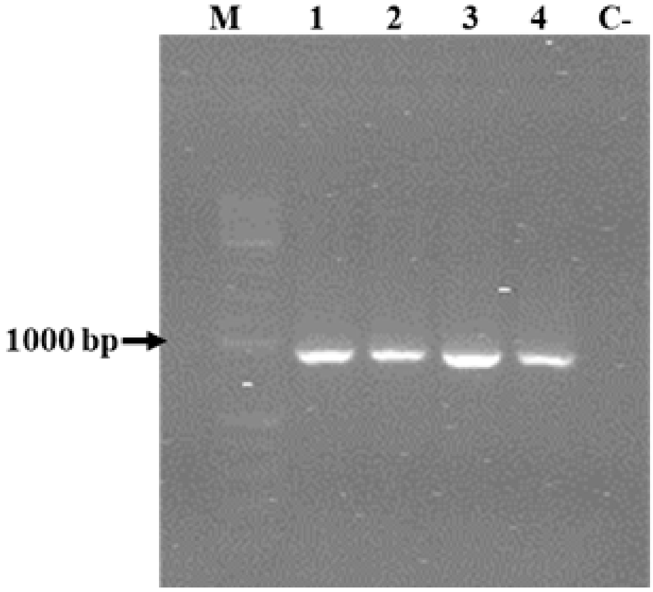

Table S1. List of primer pairs used for Phire PCR and RT-PCR experiments.

Primer	sequence	T annealing (°C)	Fragment size (bp)	References
CaMVFw<br>CaMVRev	5’>TTGGGTGGAGAGGCTATTCG>3<br>5’>CTTCCCGCTTCAGTGACAAC>3’	58	225	This work
GUSFw<br>GUSRev	5’>CGACTGGGCAGATGAACATG>3’<br>5’> TACTCCACATCACCACGCTT>3’	60	215	This work
psbAFw<br>psbArev	5’ >GAAAACCGTCTTTACATTGGA>3’<br>5’>AGTTGTGAGCATTACGTTCAT>3’	55	942	Bettini et al. 1988 [39]
